# From One-Size-Fits-All to Precision Care: Biomarker-Guided Lung Recruitment in Acute Respiratory Distress Syndrome

**DOI:** 10.7759/cureus.92984

**Published:** 2025-09-22

**Authors:** Anirban Bhattacharjee, Habib Md R Karim, Dalim K Baidya, Ankur Khandelwal, Mahammad A Aspari, Shamim Ahmad A Bhat

**Affiliations:** 1 Anaesthesiology, Critical Care, and Pain Medicine, All India Institute of Medical Sciences, Guwahati, Guwahati, IND; 2 Emergency Medicine, King Saud Medical City, Riyadh, SAU

**Keywords:** acute respiratory distress syndrome [ards], cytokine release, lung volume recruitment, personalized patient care, precision therapy

## Abstract

Acute respiratory distress syndrome (ARDS) poses the problem of ventilating a heterogeneous lung with both well-aerated and collapsed lung units with different elastances. The interfaces between open and closed alveoli are exposed to amplified stress, thereby increasing the probability of ventilator-induced lung injury (VILI). An “open lung approach” of opening closed alveoli with recruitment maneuvers (RM) and keeping them open with high positive end-expiratory pressure (PEEP) was proposed as a promising method of homogenizing gas distribution and reducing the risk of VILI. This approach has been shown to improve oxygenation consistently. However, studies have found conflicting results on the effect of RM on patient outcomes, with the latest randomized controlled trial (RCT) showing increased mortality with the use of RMs. The failure of randomized trials testing RMs may be due to the inclusion of a heterogeneous group of ARDS patients without focusing on the ARDS phenotypes most likely to benefit from RM.

Recent exploration of ARDS inflammatory phenotypes and the differential effect of therapeutics, especially high PEEP, based on phenotypes, raises the possibility of personalizing RMs to identify patient subgroups likely to benefit from the procedure. Findings of the research measuring inflammatory cytokines in bronchoalveolar lavage (BAL) fluid before and 24 hours after RMs in ARDS patients indicate the need for further research to define the role of inflammatory biomarkers in individualizing ventilation strategies in ARDS patients.

## Introduction and background

The definition of acute respiratory distress syndrome (ARDS) has evolved over the years [1]. The recent global definition built over the Berlin definition characterizes ARDS as acute onset or worsening of hypoxemic respiratory failure (PaO_2_:FiO_2_ ⩽300 mmHg or SpO_2_:FiO_2_ ⩽315 (if SpO_2_ ⩽ 97%) on high flow nasal oxygen with a flow of ⩾30 L/min or on nonivassive ventilation with at least 5 cm H_2_O end-expiratory pressure or continuous positive airway pressure and imaging showing bilateral opacities on chest radiography and CT or bilateral B lines and/or consolidations on ultrasound not fully explained by effusions, atelectasis, or nodules/masses [1,2]. The new global definition has also been adopted for resource-limited settings where neither end-expiratory pressure nor a minimum flow rate of oxygen is required for the diagnosis [1,2]. It is characterized by variable lung aeration, decreasing along the gravitational line [3]. Recruitment maneuvers (RMs) can open the non-aerated lung regions and risk inducing volutrauma in more compliant lung areas, potentially exacerbating ventilator-induced lung injury (VILI). While several studies and their meta-analyses consistently showed improvement in oxygenation, they have provided conflicting results regarding the efficacy and safety of RMs in terms of mortality benefits in ARDS [4-8].

A recent study has shown a role of cytokines, especially IL-6, in bronchoalveolar lavage (BAL) fluid in ARDS patients in determining the need for RMs and recruitability [9]. This opinion review aims to present insights into the implications of cytokine levels in BAL fluid, and the possibility of applying precision medicine of individualized RMs based on the biomarker levels. We have also concisely reviewed the physiological rationale, literature on RMs, and the scope of individualizing RMs to improve patient outcomes.

## Review

Physiological background

ARDS is defined as a clinical syndrome characterized by an acute onset of hypoxemia associated with bilateral lung infiltrates consistent with pulmonary edema in patients with pulmonary or extrapulmonary risk factors [10]. ARDS lungs become heterogeneous, with near-normal aerated alveoli in non-dependent areas and gasless, consolidated, and completely collapsed alveoli in dependent zones, separated by a zone of recruitable alveoli that open and close during each inspiration and expiration [11]. Injudicious mechanical ventilation by exposure to high transpulmonary pressure (barotrauma) and consequent overdistension (volutrauma) can lead to added insult to the inflamed lungs (ventilator-induced lung injury). Likewise, cyclical collapse and opening of recruitable alveoli can lead to further lung injury (atelectrauma) [12]. Therefore, an ideal mechanical ventilation strategy should prevent atelectrauma by maintaining adequate airway pressure while avoiding overdistension and volutrauma. It will be difficult in a heterogeneous lung with areas of different elastances.

In 1970, Mead et al. developed a mathematical model demonstrating that the interface between normal and collapsed alveoli is exposed to local stress significantly greater than the global transpulmonary pressure. Therefore, the cumulative sum of local shear stresses in a heterogeneous lung is far greater than in a homogenous lung due to the stress amplification at such interfaces of aerated and collapsed alveoli [13]. Secondly, according to Laplace’s law (Pressure=2*Tension/Radius), the driving pressure needed to open an alveolus is inversely proportional to the initial radius of the alveoli. Therefore, collapsed alveoli (with a lower initial radius) have higher opening pressure than open alveoli [14]. Thus, a heterogeneous lung is exposed to a higher risk of developing VILI due to the requirement of higher airway pressures to open the collapsed lung units and regional stress amplification. Certainly, homogenization of the lungs should achieve a more uniform stress distribution and prevent regional stress amplification, while allowing for lung-protective ventilation at lower driving pressures [15]. The current lung-protective ventilation with low tidal volumes and restrained airway pressure needs to address this problem of derecruitment and heterogeneity of lungs.

Lachmann proposed homogenizing lung ventilation by opening collapsed lung units and maintaining them in an open state [16]. The “open lung approach” involves recruiting the collapsed alveoli with RMs, followed by low tidal volume ventilation at high positive end-expiratory pressure (PEEP) to keep the recruited alveoli open [16].

Evidence summary

Multiple studies and meta-analyses found improved oxygenation and either beneficial or no effect on mortality using the 'open lung approach' and RMs [4-8]. Kacmarek and colleagues have also seen a reduction in driving pressure after administering lung RM [7]. This finding aligns with our physiological understanding that RMs enhance lung compliance.

Studies have not demonstrated a mortality benefit from RMs. Meade and colleagues randomized 983 patients with ARDS and PaO_2_:FiO_2_ <250 mmHg to an “open lung” ventilation strategy involving RMs and high PEEP versus conventional lung protective ventilation [4]. Although they found improvement in oxygenation, fewer deaths due to refractory hypoxemia, and less need for rescue therapies, the primary outcome of all-cause hospital mortality was not different between the two groups. Another multicentre randomized controlled trial (RCT) could not demonstrate improvement in 28-day mortality and ventilator-free days using the staircase RM followed by PEEP titration [17]. A watershed moment in the history of RM came when a multicentre RCT, the Alveolar Recruitment for Acute Respiratory Distress Syndrome Trial (ART), found increased mortality with an open lung approach combining staircase RMs and decremental PEEP titration compared to conventional lung protective ventilation [18].

The lack of benefit with RM and the possibility of harm led to adopting a prone position as a favored method of lung homogenization. Indeed, prone ventilation has been shown to homogenize lung gas distribution through several mechanisms discussed in an excellent review [19]. The clinical benefit of lung homogenization by prolonged proning (>12 hours) was demonstrated in the landmark Proning Severe ARDS Patients (PROSEVA) study and subsequent meta-analysis, especially in patients with moderate-severe ARDS (PaO_2_:FiO_2_ ratio <150 mmHg) [20,21]. Based on the current literature, prone positioning should always be favored over RM for moderate-severe ARDS. Further, proning should be considered early and for a prolonged period [20,21].

Nevertheless, some patients do not respond to prone ventilation. The prevalence of prone non-responders ranges from 10% to 30% [22]. Prone positioning may be non-feasible and contraindicated in some patients (unstable spinal trauma, for example). RMs may still find a place in the treatment of refractory hypoxemia in such conditions in the absence of extracorporeal therapies. Therefore, it is essential to identify the following for the judicious use of RMs: a) clinical characteristics of patients likely to respond favorably to RMs, and b) optimal method of RM to improve lung mechanics while preventing deleterious hemodynamic and pulmonary side effects.

Patient selection as a key limitation of ART

The increased mortality seen in ART with RMs is explained by the fact that the study included all ARDS patients across the severity spectrum and did not differentiate between responders and non-responders [23]. The recruitability of lungs increases with increasing severity of ARDS [24]. Therefore, RMs might be more helpful in severe ARDS than in mild to moderate ARDS. RM has been shown to lead to a threefold increase in mechanical power delivery in mild ARDS compared to severe ARDS, as reported in an exploratory analysis of the ART trial [25]. From a lung-protective ventilation standpoint, RMs in the ART cohort achieved only a ~2 cm H₂O reduction in driving pressure, indicating that most patients were likely non-recruitable [23].

Precision medicine is the way forward

As seen with the ART trial, several other therapeutic strategies failed to demonstrate benefits in randomized trials due to the inclusion of a heterogeneous group of patients with varied etiology, severity, morphology, and inflammatory status. Since the introduction of the American-European Consensus Criteria, experts have recommended that therapeutic strategies be tested on homogenous study populations based on natural history, clinical features, and biology alone or in combination [26]. While most studies have included ARDS patients across the spectrum of severity, the clinical efficacy of the ventilation strategies differs based on the severity of ARDS, as discussed below.

An analysis of ventilatory variables from a pooled database of ARDS patients enrolled in major RCTs has shown that lung compliance influences the effect of ventilation strategy on clinical outcomes [27]. In severe ARDS with poor lung compliance, minimizing driving pressure while increasing respiratory rate was associated with lower odds of death. Conversely, this approach of increasing respiratory rate is associated with higher odds of mortality in less severe ARDS with higher lung compliance [27]. Although higher versus lower PEEP did not show a mortality benefit in three large randomized controlled trials, the treatment effect varied between patients with PaO_2_:FiO_2_ above and below 200 mmHg. Higher PEEP was associated with mortality reduction in moderate-severe ARDS and increased mortality in mild ARDS [28].

Morphologically, focal and diffuse ARDS have distinct pathophysiologies and differences in response to ventilation strategies. Specifically, non-focal or diffuse bilateral ARDS is likely to benefit from higher PEEP and RMs [29]. At the same time, focal ARDS may be harmed by RMs due to overdistension of normally aerated areas [29]. A randomized trial compared a ventilatory strategy personalized based on focal and non-focal ARDS with a routine ventilation strategy in 400 patients with ARDS [30]. The use of a personalized ventilation strategy was not associated with an improvement in mortality. However, 21% of study participants were misclassified. In the subgroup of misclassified patients, mortality was substantially increased with the 'wrong' personalized ventilation strategy [28]. Therefore, personalizing ventilation to ARDS morphology may decrease mortality if the patients are correctly classified. The study also indicates the poor reliability of morphological phenotyping of ARDS with radiographs or CT scans.

Another way of estimating lung recruitability is by measuring the Recruitment-to-Inflation (R/I) ratio [31]. Patients with a high R/I ratio (i.e., higher recruitability) show improved oxygenation and compliance after RM without a reduction in pulse pressure [31,32]. Moreover, there were higher hemodynamic perturbations with reduced pulse pressure with RMs in patients with a low R/I ratio [29,30]. Another study has shown that the PEEP-induced increase in pulmonary vascular resistance happens only in lungs with a low R/I ratio [33]. The problem with the R/I ratio is that the bedside measurement of the R/I ratio with modern ventilators has an error margin of 10-20% [34]. Therefore, using a single cutoff of 0.5 to differentiate recruitable from non-recruitable lungs may be erroneous. Several methods are used to assess recruitability in ARDS patients, each with its own strengths and limitations (Table 1). 

**Table 1:** Different methods used for recruitability assessment in ARDS patients* ^*^[35-43] ARDS: acute respiratory distress syndrome; PEEP: positive end-expiratory pressure

Procedure	Strengths	Limitations
PEEP up titration with assessment of change in compliance	It can be performed bedside with patient transport. Does not require costly equipment. Usually coupled with a change in oxygenation and hemodynamic parameters to arrive at optimal PEEP, and therefore, a more clinically oriented method	Serial bedside assessments by skilled intensivists are required. No robust evidence of superiority over the ARDS PEEP-FiO_2_ table in terms of patient outcomes
Assessment by computed tomography (CT) [35]	Provides an anatomical picture of lung units that were recruited after administration of higher PEEP. Quantitative measurement of recruitability can be obtained as a percentage of total lung volumes	Requires patient transport to CT rooms. Costly and involves exposure to ionizing radiation. Serial measurements are often not feasible. CT scan accounts for only the previously collapsed units that opened with high PEEP, and not the already open units that overdistended with high PEEP
Lung ultrasound [36]	Non-invasive and repeatable. It can be performed bedside without patient transport. Correlates with lung mechanics-based recruitability assessments	Requires expertise for serial monitoring. Does not assess PEEP-induced overdistension and, therefore, requires complementary alternate methods
Electrical impedance tomography (EIT) [37]	Non-invasive and radiation-free. Provides real-time and continuous assessment of ventilation distribution and its change due to alteration in PEEP and clinical evolution. Allows assessment of the recruitability of lungs and determination of optimal PEEP	Costly and not easily available. Robust evidence in favour of EIT-guided ventilation is lacking
Static pressure–volume (P–V) curves [38-40]	The shape of the P-V inflation curve, the area of the hysteresis loop, and compliance above lower inflection points may be used as a surrogate for the estimation of lung recruitability	Requires disconnection from the ventilator if the “supersyringe” method is used, although a “constant flow” method can produce identical P-V loops without disconnection. Quasi-static methods require specialised algorithms in ventilators. Requires deep sedation and muscle relaxation and cannot be performed in spontaneously breathing patients
End expiratory lung volume (EELV) measurement [41,42]	EELV was measured by the modified nitrogen wash-in/wash-out method bedside without ventilator disconnection. Correlates with CT-based measurements of EELV	Not widely available in many centres. An increase in EELV with PEEP cannot be estimated due to overdistension of already aerated lung units
Recruitment-inflation ratio [32,43]	Can be easily estimated bedside by a PEEP drop from 15 cm H_2_O to 5 cm, and calculation with an online calculator (https://rtmaven.com/ri-ratio). Easy interpretation with dichotomous result: An R/I ratio more than 0.5 suggests potential for lung recruitment compared to overinflation with higher PEEP. High R/I ratio correlates with improvement in oxygenation and respiratory compliance with recruitment manoeuvres	It is unclear how optimal PEEP can be set based solely on the R/I ratio. Recruitability may be non-uniform across the 10 cm H_2_O PEEP drop; a more granular R/I ratio may measure recruitability more precisely

Role of inflammatory biomarkers in individualizing ARDS management

Biologic phenotyping holds promise mainly because the biologic markers precede clinical expression and may facilitate earlier and more precise identification of different host-response features that influence clinical outcomes and responses to therapeutics [44].

**Figure 1:**
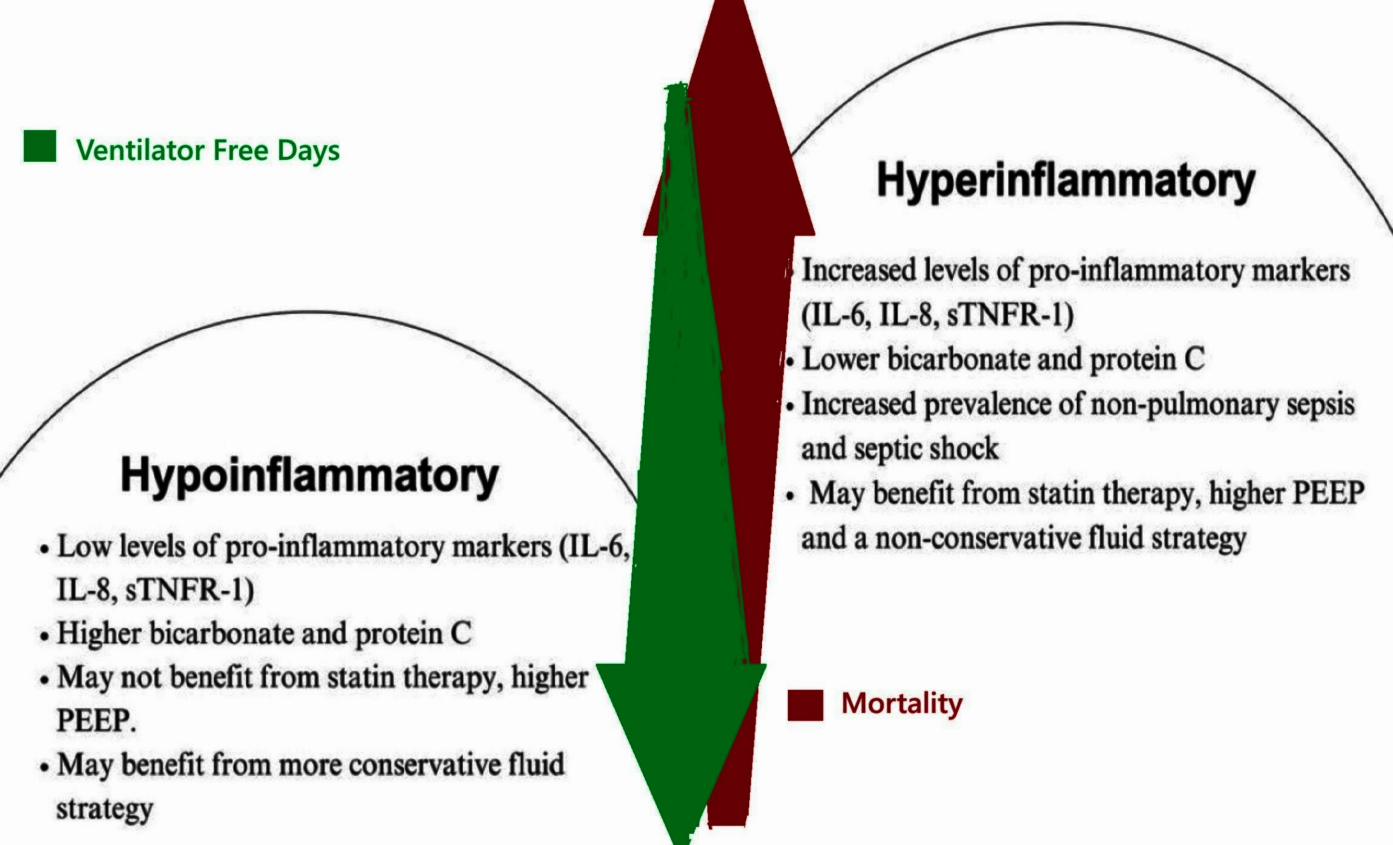
Relationship of inflammatory marker levels and patient outcome in terms of mortality and ventilator-free days IL: interleukin; PEEP: positive end-expiratory pressure; TNFR: tumor necrosis factor receptor superfamily

Calfee and colleagues used a statistical method called latent class analysis to identify two different phenotypes of ARDS within the heterogeneous study population enrolled in the Alveolar Recruitment and Mechanical Ventilation (ARMA) and Assessment of Low tidal Volume and elevated End-expiratory volume to Obviate Lung Injury (ALVEOLI) trials (Figure 1) [45].

The hyperinflammatory phenotype includes patients with increased inflammatory markers, circulatory shock, lower plasma bicarbonate levels, and a higher mortality rate compared to the hypoinflammatory phenotype. These two phenotypes were also found in subsequent analyses of other ARDS cohorts [46,47]. The "hyperinflammatory" phenotype patients responded favorably to high PEEP, while the "hypoinflammatory" phenotype patients did not [45]. Although the use of Simvastatin was not associated with a significant difference in clinical outcome in the HARP-2 study [48], another subsequent latent class analysis found significant improvement in 28-day mortality with the use of Simvastatin in the “hyperinflammatory” phenotype [49]. A conservative fluid management strategy is associated with a reduction in mortality in hypoinflammatory ARDS but may increase mortality in hyperinflammatory ARDS [47].

Can inflammatory biomarkers identify patients who can benefit from RMs?

From the above discussion, it is evident that the response to ventilator strategy may differ based on the clinicobiological phenotype of ARDS. The beneficial effect of PEEP in ARDS of hyperinflammatory phenotype indicates that the lungs in hyperinflammatory ARDS are more recruitable and benefit from RMs and an 'open lung' approach.

A secondary analysis of patients recruited in the Permissive Hypercapnia, Alveolar Recruitment and Low Airway Pressure (PHARLAP) trial, which compared RMs and standard treatment, found a trend toward increased ventilator-free days using RMs and high PEEP in the hyperinflammatory phenotype [50]. However, the interaction of phenotype and treatment effect was not statistically significant. In both the inflammatory phenotypes, cytokine levels were decreased in serum and BAL fluid using RMs [38]. Such a reduction in inflammatory cytokine levels with RM points towards the possibility of a decrease in biotrauma with RM.

Recently, Shen and Huai tested the strategy of identifying ARDS patients likely to benefit from RM. Specifically, they measured the concentrations of IL-6, IL-8, and IL-10 in BAL fluid and assessed lung ultrasound score (LUS) before and 24 hours after lung recruitment in 62 mechanically ventilated patients with ARDS [9]. The study population was moderately hypoxaemic with a mean PaO_2_:FiO_2_ ratio of 119 mmHg. The authors classified patients into RM-effective and RM-non-effective groups based on the LUS score changes, though the specific cutoff for this classification is unclear. The baseline IL-6 levels in BALF correlated moderately with LUS; the correlation of other biomarkers with LUS was weak. The study has shown that lung recruitment was associated with reduced BAL cytokine levels in both RM-effective and RM-non-effective groups. A similar reduction of inflammatory markers with RMs was reported in another study [38]. A plausible explanation for the decrease in inflammatory markers is the reduction in cyclical collapse and opening of alveoli (atelectrauma) and reduction in cumulative stress by homogenization of the lung (as explained above). The decrease in BALF IL-6 levels was significantly greater in the RM-effective versus the RM-non-effective group. This finding indicates that the degree of change in inflammatory biomarkers may indicate the recruitability of the lungs.

Other biomarkers in ARDS

A good number of other biomarkers for both diagnostic and prognostic purposes are described for ARDS [51]. Over and above the cytokines and chemokines, products arising during the exudative phase of ARDS from endothelial cell damage, alveolar cell damage, and vascular permeability can also act as biomarkers; angiopoietin-1/2, endothelin, surfactant proteins-B/D, Krebs von den Lungen-6 (KL-6), soluble receptor for advanced glycation end-products (sRAGE), lactate dehydrogenase (LDH), von Willebrand factor (vWF) are a few them [52]. Although a meta-analysis showed the diagnostic use of LDH, sRAGE, and vWF for ARDS in high-risk patients, the results must be further ascertained [52,53]. Higher levels of endothelial-derived protein (i.e., angiopoietin-2) were noted in medical and surgical critical care patients who developed ARDS [54,55]. Plasma KL-6 levels are elevated in patients with ARDS and correlate with mortality [56].

Research gaps

Shen and Huai's study demonstrated a reduction in inflammatory biomarkers with RM, and the decrease was more significant in patients where RM improved lung aeration, as seen in the ultrasound [9]. However, the study did not show the relationship between changes in biomarkers and clinical outcomes. Although the reduction of biomarkers was greater in the RM-effective group, the oxygenation improvement did not vary significantly between the two groups. It remains to be seen if BALF cytokine-guided patient selection for RM improves clinical outcomes. Future research should focus on studying the performance of the BAL biomarker change in patients likely to benefit from RM. Shen and Huai's study used sustained inflation to a mean airway pressure of 40 cm H_2_O for 30 seconds as the method for lung recruitment [9]. In recent years, sustained inflation for more than one minute has fallen out of favor due to the increased risk of hemodynamic instability, barotrauma, and cardiac arrest. Briefer inflation (defined as duration <60 seconds) may also produce transient hemodynamic instability in the presence of concomitant heart failure [57]. More recently, a ‘staircase’ increase in airway pressure/PEEP to slowly increase transpulmonary pressure has achieved equivalent recruitment as sustained inflation with lower airway pressure [58].

## Conclusions

In the current era of precision medicine, it is crucial to recognize that ARDS is a heterogeneous syndrome with varied etiopathology, clinical features, lung mechanics, and response to treatment. Clinical management varies according to the severity of ARDS, respiratory compliance, and levels of inflammation. So far, we have indications that the hyperinflammatory ARDS, diffuse bilateral ARDS, with increased severity of hypoxemia and poor lung compliance, are more likely to respond favorably to RMs and higher PEEP. The potential harm of RMs, such as increased lung damage, might be mitigated by identifying the ARDS phenotypes most likely to benefit from these interventions based on clinical characteristics and lung mechanics. Although secondary analyses of existing data are encouraging, it remains to be seen if a ventilation strategy individualized using inflammatory markers improves clinical outcomes in future randomized trials.
